# Enhancement of the technical and non-technical skills of nurse anesthesia students using the Anesthetic List Management Assessment Tool in Iran: a quasi-experimental study

**DOI:** 10.3352/jeehp.2023.20.19

**Published:** 2023-06-16

**Authors:** Ali Khalafi, Maedeh Kordnejad, Vahid Saidkhani

**Affiliations:** Department of Anesthesiology, School of Allied Medical Sciences, Ahvaz Jundishapur University of Medical Sciences, Ahvaz, Iran; Hallym University, Korea

**Keywords:** Anesthetics, Clinical competence, Formative feedback, Iran, Nursing assessment

## Abstract

**Purpose:**

This study investigated the effect of evaluations based on the Anesthetic List Management Assessment Tool (ALMAT) form on improving the technical and non-technical skills of final-year nurse anesthesia students at Ahvaz Jundishapur University of Medical Sciences (AJUMS).

**Methods:**

This was a semi-experimental study with a pre-test and post-test design. It included 45 final-year nurse anesthesia students of AJUMS and lasted for 3 months. The technical and non-technical skills of the intervention group were assessed at 4 university hospitals using formative-feedback evaluation based on the ALMAT form, from induction of anesthesia until reaching mastery and independence. Finally, the students’ degree of improvement in technical and non-technical skills was compared between the intervention and control groups. Statistical tests (the independent t-test, paired t-test, and Mann-Whitney test) were used to analyze the data.

**Results:**

The rate of improvement in post-test scores of technical skills was significantly higher in the intervention group than in the control group (P˂0.0001). Similarly, the students in the intervention group received significantly higher post-test scores for non-technical skills than the students in the control group (P˂0.0001).

**Conclusion:**

The findings of this study showed that the use of ALMAT as a formative-feedback evaluation method to evaluate technical and non-technical skills had a significant effect on improving these skills and was effective in helping students learn and reach mastery and independence.

## Graphical abstract

[Fig f2-jeehp-20-19]

## Introduction

### Background/rationale

Evaluation is an essential component of any educational process, and it provides evidence for students’ achievement of learning goals [1]. If an evaluation is accompanied by appropriate feedback, it can best improve the learner’s skills [2]. A professional evaluation of skills in healthcare settings entails evaluating both technical and non-technical skills [3]. Nurse anesthetists play an essential role in providing anesthesia care and dealing with anesthesia-related complications [4]. Traditional clinical education that is currently offered in hospitals often cannot create maximally effective learning opportunities [5]. A formative evaluation facilitates teaching and, consequently, learning [6]. Given the important role of clinical students in the safe care of patients, it is necessary to evaluate these students’ qualifications based on clear professional standards [7]. However, in order to improve services and reduce mistakes in sensitive environments such as the operating room, it is particularly important to pay attention to non-technical skills [8]. Therefore, it is now considered important to use more comprehensive methods to measure all the skills needed to provide care as part of an anesthesia team [9]. One of the new clinical evaluation methods used for assessing cognitive knowledge, as well as technical and non-technical skills, is the Anesthetic List Management Assessment Tool (ALMAT). The Royal College of Anesthetists developed the ALMAT evaluation method based on the Acute Care Assessment Tool evaluation method [10]. This evaluation model is designed to evaluate the cognitive knowledge of anesthesia residents and facilitate feedback on technical and non-technical skills [11]. ALMAT is a clinical and performance-based evaluation method, the goal of which is to bring students to competence and independence. For this reason, it is suitable for final-year students [10]. No study has yet been conducted on this evaluation method, and it has only been used as an educational-evaluation guideline at one of the British Royal Colleges for the training and evaluation of final-year anesthesia residents [11].

### Objectives

We hypothesized that compared to common educational methods, the implementation of evaluation based on the ALMAT form as a formative-feedback evaluation method would improve technical and non-technical skills, and help learners achieve mastery and independence.

## Methods

### Ethics statement

This study was approved by the Ethics Committee of AJUMS (Ref. ID: IR.AJUMS.REC.1401.476). Informed consent was obtained from participants.

### Study design

This was a quasi-experimental study, with a non-equivalent control group pre- and post-test design. It was described according to the Transparent Reporting of Evaluations with Nonrandomized Designs statement.

### Setting

This study was conducted at 4 university hospitals (Golestan Hospital, Imam Hospital, Razi Hospital, Taleghani Hospital) affiliated with AJUMS during 1 academic semester (October, November, and December 2022). Weekly observations and feedback based on ALMAT (Supplement 1) to improve the technical and non-technical skills of senior students (last year of the bachelor’s degree program) continued until the students achieved mastery and independence in the targeted skills. To evaluate the effect of ALMAT on the improvement of students’ technical and non-technical skills, pre- and post-tests were used (Supplements 2, 3). Before the implementation of the intervention, both groups of students (the intervention group and the control group) took a pre-test, and after the last formative assessment using the ALMAT method in the intervention group, a post-test was conducted for both groups.

### Participants

**Fig. 1.**
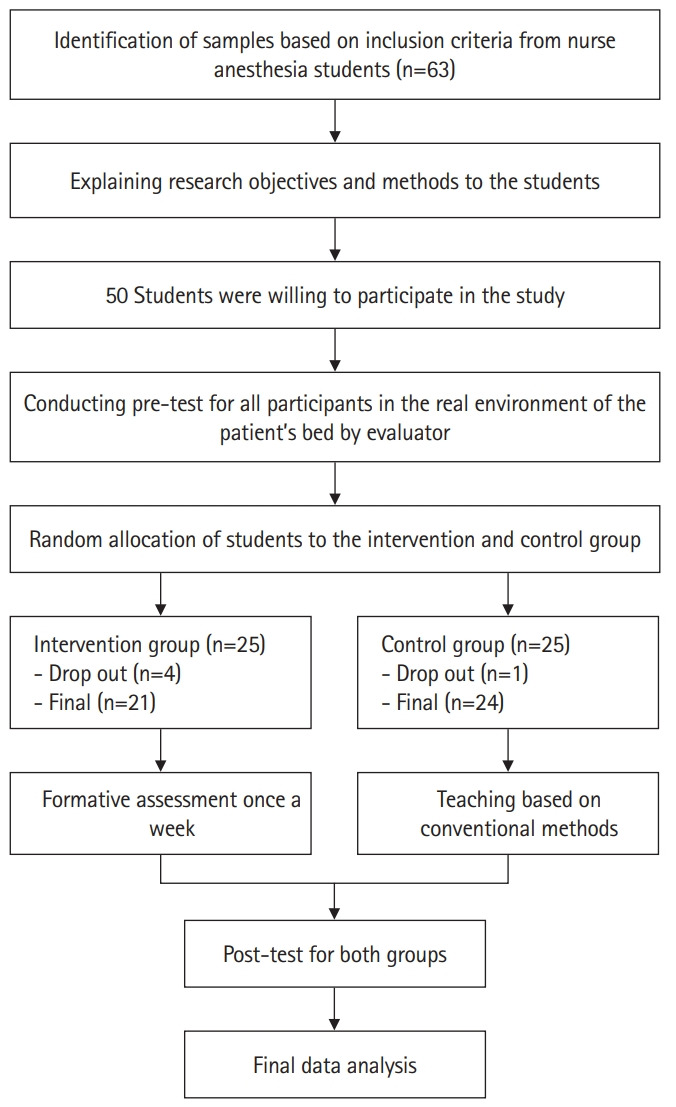
Flowchart of the study.

All the final-year nurse anesthesia students (n=63) of AJUMS were invited to take part in this study. Of these, 50 agreed to participate in this study and signed a written informed consent form. As the study proceeded, 5 students dropped out: 1 from the control group and 4 from the intervention group. For this reason, only the data of the students who completed the pre- and post-test were analyzed (Fig. 1).

### Interventions

At first, a pre-test was conducted to evaluate the students’ technical and non-technical skills separately in both the intervention and control groups. The next step was the selection of instructors. At each of the 4 hospitals under study, a nurse anesthesia instructor with more than 5 years of experience in the clinical training of nurse anesthetists was selected. Then, all 4 instructors participated in a briefing session with the members of the research team and were acquainted with the objectives of the study, the ALMAT form, its dimensions, its items, and how to provide oral and written feedback. Each of the instructors performed 2 observations on 2 students’ performance and gave them feedback, and then the problems and ambiguities were resolved. Afterwards, the intervention started and the students in the intervention group received a formative assessment based on the ALMAT form. In this way, the performance of each student during the induction of general anesthesia was carefully examined and observed once a week by the instructor based on the ALMAT form, which included technical and non-technical items, and then verbal feedback was immediately given to the student with full details. Additionally, for more durability and impact, the feedback given to each student was carefully recorded in the ALMAT form related to that particular student, and a copy was also provided to the student. In each observation, the level of supervision required by students was determined by the instructors based on their performance (using a range of 1, 2A, 2B, 3, 4, and not applicable [N/A]), and the evaluation continued until the students reached the N/A level of mastery and independence in learning (Supplement 1). Feedback was given to the students based on the strengths and weaknesses of their performance in these areas. The frequency of observations and feedback in the intervention group varied from 6 to 10 times based on the rate and pace of their progress towards reaching the N/A level. The control group was trained in the traditional way and did not receive regular feedback until the student reached independence. In this group, the same training method and evaluation method that the trainer had used before was implemented. After the last ALMAT formative assessment was carried out in the intervention group (i.e., after the students achieved independence from their instructor’s point of view), the post-test was conducted for both groups with the same checklists used in the pre-test.

### Outcomes

The outcomes of this study included students’ technical and non-technical skills before and after the ALMAT assessment and their technical and non-technical skills before and after clinical training based on the conventional method.

### Data sources/measurement

The Anesthetists’ Non-Technical Skills (ANTS) standard checklist, which is designed to check the non-technical skills of anesthesia team members, was used. This instrument was developed by Flin et. [12] in 2000 in Scotland. It has 15 items in 4 areas (task management, team working, situational awareness, and decision-making). This questionnaire is scored based on a 5-point Likert scale, with 5, 4, 3, 2, and 1 representing the options “good,” “acceptable,” “moderate,” “weak,” and “not observed,” respectively (Supplement 2). ANTS is a valid and reliable standard tool that has been psychometrically analyzed. The results of factor analysis have formed the tool into 4 subscales. Cronbach’s α coefficient for the first, second, third, and fourth subscales was 0.89, 0.73, 0.88, and 0.82, respectively. The internal consistency of this instrument has been reported to be good. Furthermore, significant and strong correlations have been observed among the 4 subscales of the tool [12,13]. The second instrument included a researcher-made checklist used to measure technical skills. This checklist measured the psychomotor skills of nurse anesthesia students in anesthesia care, with 19 items in 3 subscales (before induction, during induction, and after induction). It was scored based on a 5-point Likert scale with the options “good,” “acceptable,” “moderate,” “weak,” and “not observed” represented by 5, 4, 3, 2, and 1, respectively (Supplement 3). The content validity of this checklist was confirmed by seeking the opinion of 10 faculty members of the Department of Anesthesiology of AJUMS. In order to check the reliability of the tools, the internal consistency method was used, and a Cronbach’s α coefficient of 0.88 was obtained.

### Bias

In order to prevent differences in evaluators’ performance and to keep evaluations consistent, a briefing session was held for all evaluators participating in the study about the evaluation checklists and how to complete them, and an already completed form was provided to the evaluators. In order to calculate inter-rater reliability, all evaluators observed and evaluated a similar case in the presence of researchers, then they received feedback on how they made their evaluation, and the possible problems were eliminated.

### Study size

Based on similar studies, the sample size calculation was conducted using G*Power ver. 3.1.9.2 (Heinrich-Heine-Universität Düsseldorf; http://www.gpower.hhu.de/), based on the independent sample Student t-test, a 2-tailed alpha of 0.05, power (1-β) of 0.8, and a large effect size of 0.8. The result showed that a sample size of 25 per group was required. Our study included 25 students in the control group and 25 students in the intervention group.

### Assignment method

All final-year nurse anesthesia students who participated in the study were selected using the census method and were randomly assigned to the intervention and control groups. Randomization was done through a table of random numbers.

### Blinding (masking)

No blinding was done.

### Unit of analysis

The unit of analysis was the group (experimental or control).

### Statistical methods

IBM SPSS ver. 25.0 (IBM Crop.) was used for data analysis. Data analysis included descriptive and inferential statistics. The latter involved comparing the mean of quantitative variables in the 2 groups using the independent t-test and paired t-test, and if the assumptions of the test were not established, its non-parametric equivalent (i.e., the Mann-Whitney test), was used. The significance level was set at P<0.05.

## Results

### Participants

Fifty nurse anesthesia students including 8 men (17.8%) and 37 women (82.2%) participated in this study, and there were 5 dropouts (Table 1).

### Technical skills of nurse anesthesia students

The results of the study showed that the mean scores for technical skills in the post-test increased significantly compared to the pre-test (55.76±4.11 versus 87.76±2.52, P<0.0001). In the post-test, a statistically significant difference was observed between the intervention and control groups (P<0.0001). Before the intervention, a comparison of the mean scores of the technical skills and their dimensions showed no significant difference between the control and intervention groups. It can be concluded that the use of ALMAT formative-feedback assessment had a significant effect on the improvement of the technical skills of nurse anesthesia students (Tables 2, 3). Raw response data of participants for each item of the pre- and post-test for technical skills is available from Dataset 1.

### Non-technical skills of nurse anesthesia students

The mean scores of non-technical skills in the post-test increased significantly compared to the pre-test (31.57±3.17 versus 2.73±66.42, P>0.0001). In the post-test, a statistically significant difference was observed between the intervention and control groups (P>0.0001). Before the intervention, a comparison of the mean scores of non-technical skills and their dimensions showed no significant difference between the control and intervention groups. It can be concluded that the use of the ALMAT formative-feedback assessment had a significant effect on the improvement of nurse anesthesia students’ non-technical skills (Tables 4, 5). Raw response data of the participants for each item of the pre- and post-test for non-technical skills is available from Dataset 2.

## Discussion

### Key results

This study investigated the effect of ALMAT evaluations on the improvement of technical and non-technical skills of nurse anesthesia students. Based on the findings of the present study, the intervention group showed a greater degree of improvement in technical and non-technical skills than the control group, and the hypothesis of the study was confirmed.

### Interpretation

The results of the study showed that the use of ALMAT for formative assessments can accelerate mastery and independence in these skills among final-year students. Furthermore, according to the students’ scores, the use of ALMAT promoted student learning. In fact, by virtue of this assessment method, students tend to identify their strengths and weaknesses, and by trying to enhance the former and eliminate the latter, they prepare themselves to enter the clinical setting. In addition, given the fact that the students received feedback in the real environment immediately after their performance, and that these evaluations were repeated on a weekly basis, it was substantially easier for the students to move towards mastery and independence. This also contributed to the better retention of the educational points resulting from the feedback given. It should be noted that the rate of using these points in the next similar situation was higher than when these points were presented only in the classroom or at the end of an internship program. Due to the fact that the effectiveness and quality of teaching methods are usually measured by the results of students’ performance, it is necessary for managers in the field of clinical education of students to adopt appropriate methods to improve their education and evaluation. Therefore, innovative models should be used based on formative feedback such as ALMAT for the clinical training of learners [11].

### Comparison with previous studies

To the best of our knowledge, based on a review of the literature, no prior studies investigated ALMAT in detail. However, this method can be compared with similar formative-feedback evaluation methods. In line with the findings of the present study, a study in India on the use of mini-Clinical Evaluation Exercise (CEX) as a method to evaluate the technical skills of anesthesia graduates showed positive results. Most of the students and professors had positive opinions about various aspects of mini-CEX, such as its easy implementation and positive educational impact [14]. Another study in Australia was conducted on formative assessments with feedback during final-year students’ internships. In this study, 86% of the students considered this method of evaluation to be useful, and they were able to achieve independence in technical skills [15]. The results of a similar study that was conducted in Iran on the effect of oral feedback on the learning outcomes of nursing students were consistent with the results of the present study. Students in the intervention group had higher scores than the control group [7]. Again, to the best of our knowledge based on a review of the literature, no studies contradicting the results of the present study were found.

### Limitations

Since in the present study, evaluations were performed in a real clinical environment, it was not possible to completely homogenize the clinical cases. Further studies with a larger sample size could better show the effectiveness of this evaluation method.

### Generalizability

The results of this study can be useful for clinical educators to better train and evaluate students in hospitals throughout the world.

### Suggestions

The presence of nurse anesthetists is necessary during the entire anesthesia procedure in order to perform various tasks. Therefore, it is very important for them to receive accurate training and evaluations. ALMAT could be compared with other evaluation methods in terms of effectiveness.

### Conclusion

Using ALMAT for the formative assessment of students in clinical settings had a significant effect on improving their technical and non-technical skills. This model is recommended to be used as a low-cost and effective method for teaching and evaluating. It can also be used along with other educational methods for a better understanding of the strengths and weaknesses of students at the bedside.

## Figures and Tables

**Figure f2-jeehp-20-19:**
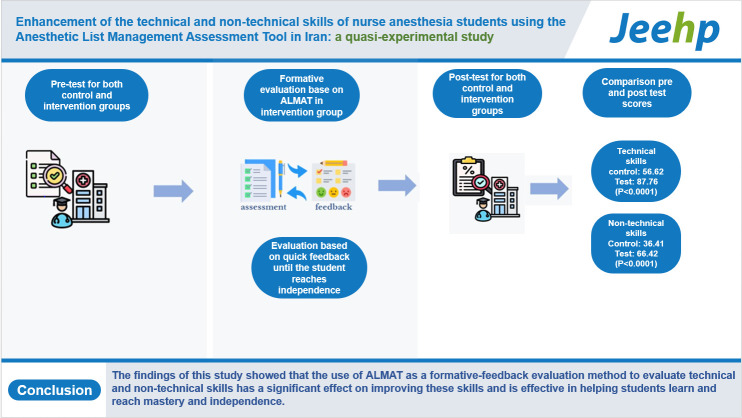


**Table 1.** Frequency distribution of demographic characteristics of the students in the intervention and control groups

Characteristic	Frequency of students		%
	Control	Test	
Gender			
Female	17	20	82.2
Male	7	1	17.8
Marital status			
Married	2	3	11.1
Single	22	18	88.9
Total	24	21	45

**Table 2.** Comparison of the average improvement in technical skills and their dimensions in the control and intervention groups before and after the intervention (independent t-test)

Variable	Pre-test			Post-test		
	Frequency	Mean±SD	P-value	Frequency	Mean±SD	P-value
Technical skills			0.830			0.0001
Control	24	55.66±5.92		24	56.62±5.49	
Test	21	55.76±4.11		21	87.76±2.52	
Before induction			0.766			0.0001
Control	24	20.37±3.42		24	21.10±2.63	
Test	21	20.09±2.73		21	32.71±1.58	
During induction			0.581			0.0001
Control	24	8.00±1.61		24	8.45±0.97	
Test	21	8.33±2.37		21	13.42±1.07	
After induction			0.711			0.0001
Control	24	27.29±4.15		24	27.00±4.34	
Test	21	26.90±2.44		21	41.61±1.43	

SD, standard deviation.

**Table 3.** Comparison of the mean and standard deviation of the pre-test and post-test scores for technical skills and their dimensions in the control and intervention groups (paired t-test)

Variable	Pre-test	Post-test	t-value	P-value
Technical skills				
Control	55.66±5.92	56.62±5.49	-0.554	0.585
Test	55.76±4.11	87.76±2.52	-33.433	0.0001
Before induction				
Control	20.37±3.42	21.10±2.63	-1.006	0.325
Test	20.09±2.73	32.71±1.58	-16.872	0.0001
During induction				
Control	8.00±1.61	8.45±0.97	-1.111	0.278
Test	8.33±2.37	13.42±1.07	-9.875	0.0001
After induction				
Control	27.29±4.15	27.00±4.34	-0.240	0.813
Test	26.90±2.44	41.61±1.43	-26.834	0.0001

Values are presented as mean±standard deviation, unless otherwise stated.

**Table 4.** Comparison of the average improvement of non-technical skills and their dimensions in the control and intervention groups before and after the intervention (independent t-test)

Variable	Pre-test			Post-test		
	Frequency	Mean±SD	P-value	Frequency	Mean±SD	P-value
Non-technical skills			0.0001			0.0001
Control	24	35.75±4.55		24	36.41±4.35	
Test	21	31.57±3.17		21	66.42±2.73	
Task management			0.007			0.0001
Control	24	9.12±1.48		24	9.00±1.88	
Test	21	7.90±1.37		21	17.95±0.38	
Teamwork			0.010			0.0001
Control	24	12.29±2.42		24	12.54±2.34	
Test	21	10.57±1.74		21	22.19±0.51	
Situational awareness			0.228			0.0001
Control	24	7.00±1.10		24	7.20±1.21	
Test	21	6.57±1.24		21	13.61±1.02	
Decision making			0.066			0.0001
Control	24	7.33±1.65		24	7.66±1.27	
Test	21	6.62±1.12		21	12.66±1.85	

SD, standard deviation.

**Table 5.** Comparison of the mean and standard deviation of the pre-test and post-test scores for non-technical skills and their dimensions in the control and intervention groups (paired t-test)

Variable	Pre-test	Post-test	t-value	P-value
Non-technical skills				
Control	35.75±4.55	36.41±4.35	-0.532	0.006
Test	31.57±3.17	66.43±2.73	-40.023	0.0001
Task management				
Control	9.12±1.48	9.00±1.88	0.257	0.799
Test	7.90±1.37	17.95±0.38	-33.874	0.0001
Teamwork				
Control	12.29±2.42	12.54±2.34	-0.370	0.715
Test	10.57±1.74	22.19±0.51	-32.122	0.0001
Situational awareness				
Control	7.00±1.10	7.20±1.21	-0.541	0.594
Test	6.57±1.24	13.61±1.02	-17.652	0.0001
Decision making				
Control	7.33±1.65	7.66±1.27	-0.984	0.335
Test	6.62±1.12	12.66±1.85	-12.680	0.0001

Values are presented as mean±standard deviation, unless otherwise stated.

## Footnotes

**Data availability**
Data files are available from Harvard Dataverse: https://doi.org/10.7910/DVN/SZYZ45 Dataset 1. Raw response data of participants for each item of the pre- and post-test (technical skills).

Dataset 2. Raw response data of the participants for each item of the pre- and post-test (non-technical skills).

## Supplementary materials

Supplementary files are available from Harvard Dataverse: https://doi.org/10.7910/DVN/SZYZ45

Supplement 1. A checklist to implement the intervention: formative assessment of students based on the using Anesthetic List Management Assessment Tool method.

Supplement 2. Pre- and post-test questionnaire on non-technical skills.

Supplement 3. Pre- and post-test questionnaires on technical skills.

Supplement 4. Audio recording of the abstract.
